# Multiple endocrine neoplasia type 1 with suspicion of Zollinger Ellison syndrome in a family with history of renal stones and hypercalcemia in a limited resource setting: a case report

**DOI:** 10.1093/omcr/omac094

**Published:** 2022-09-26

**Authors:** Kamran Hussain, Jebun Nahar, Fakhar Abbas, Jasir Nawar, Ayush Anand

**Affiliations:** Department of Internal Medicine, Jamaica Hospital Medical Center, New York, USA; Department of Internal Medicine, Jamaica Hospital Medical Center, New York, USA; Department of Internal Medicine, Jamaica Hospital Medical Center, New York, USA; Bronx High School of Science, New York, USA; B. P. Koirala Institute of Health Sciences, Dharan, Nepal

## Abstract

Multiple endocrine neoplasia type 1 (MEN 1) syndrome is a rare autosomal dominant endocrine tumour syndrome, which can be diagnosed clinically based on family history and the existence of MEN 1-associated tumours or molecularly based on genetic testing. We described the case of a Hispanic 55-year-old male presenting with dysphagia, chest pain and diarrhoea for three months with a family history of hypercalcaemia and nephrolithiasis in first-degree relatives. Primary hyperparathyroidism was suggested by hypercalcaemia, elevated parathyroid hormone level, hypercalciuria, nephrolithiasis on abdominal computed tomography scan and enlarged parathyroid gland on computed tomography pulmonary angiogram. Also, patient had hypergastrinemia and a hypodense lesion in the pancreas on computed tomography scan of abdomen. These findings suggested MEN 1 syndrome with high suspicion of associated Zollinger Ellison syndrome. Our case highlights the importance of family history and high clinical suspicion in patients presenting with primary hyperparathyroidism and hypergastrinemia.

## INTRODUCTION

Multiple endocrine neoplasia type 1 (MEN 1) syndrome, also known as Wermer syndrome, is an autosomal dominant endocrine tumour syndrome caused by mutation in the Menin 1 gene having a prevalence of 1 in 20 000 to 40 000 people [1]. It can be diagnosed clinically or by genetic testing [2]. The treatment of MEN 1 syndrome involves medical and surgical approaches and varies depending on the location of tumours. For parathyroid tumours, subtotal or total thyroidectomy, concurrent transcervical thymectomy and autotransplantation can be done [2]. Curative surgery is required for pancreatic tumours more than >1 cm in size or tumours showing aggressive growth [2]. And, for non-resectable or inoperable pancreatic tumours, somatostatin analogues, biotherapy, radiotherapy and chemotherapy can be done [2]. The tumours of pituitary can be treated by medical therapy (depending upon the type of pituitary cells involved), transsphenoidal surgical hypophysectomy and radiotherapy [2]. Thymic and bronchopulmonary tumours should be cured by surgery [2]. Gastrinoma tumours, if large, may require total gastrectomy [2]. For adrenal tumours, surgical approach is required for functioning tumours and non-functioning tumours showing aggressive growth [2]. Herein, we describe a case of MEN1 with significant family history, renal stones and tumour in the pancreas.

## CASE REPORT

A 55-year-old Hispanic male presented with complaints of dysphagia, chest pain and passage of loose stools for the past three months. Dysphagia was gradually worsening, more with solids than liquids, and associated with nausea and vomiting (five to six times per day, containing undigested food). He experienced choking spells that lasted several minutes. The patient also complained of recurrent burning chest pain, waxing and waning intensity, aggravated after having a meal, lying down and coughing. Also, he passed loose stools, semi-solid consistency, normal colour and 5–6 times a day. During the last three months, he lost 20 pounds weight. He had multiple hospital visits during the same period for these complaints.

His past history was significant for asthma, a recent episode of pneumonia and urinary tract infection. He had a habit of cigarette smoking, alcohol consumption and excess coffee intake. His family history was remarkable for hypercalcaemia and nephrolithiasis in his father and two brothers.

Initial laboratory investigations (Table 1) showed hypercalcemia, elevated parathyroid hormone (PTH), hypergastrinemia, increased urinary calcium/creatinine ratio and calcium oxalate crystals in urinalysis.

**Table 1:** Laboratory investigations of the patient

Investigations	Result	Reference range
Serum calcium (mg/dl)	11.9	8.4–10.2
Parathyroid hormone (pg/ml)	114	14–64
Vitamin D 25 hydroxy (ng/ml)	29.8	30–100
Parathyroid hormone-related peptide (pg/ml)	9	11–20
Prolactin (ng/ml)	12.7	3.7–17.9
Urinary calcium/creatinine ratio	0.31	< 0.14

Esophagogastroduodenoscopy showed Los Angeles (LA) Grade D oesophagitis (one or more mucosal breaks involving 75% of oesophageal circumference) with bleeding 40 cm from the incisors. Oesophageal biopsy showed fragments of granulation tissue with marked acute and chronic inflammation and acute inflammatory exudates.

**Figure 1:**
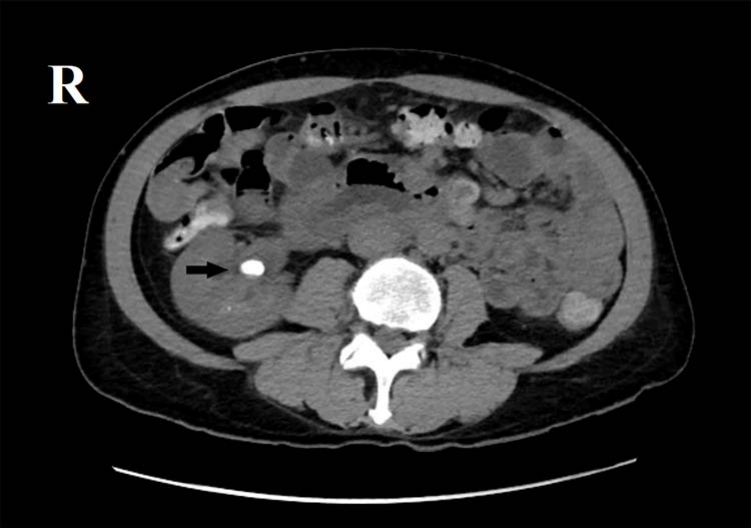
CT Scan of abdomen showing right renal pelvis calculus.

**Figure 2:**
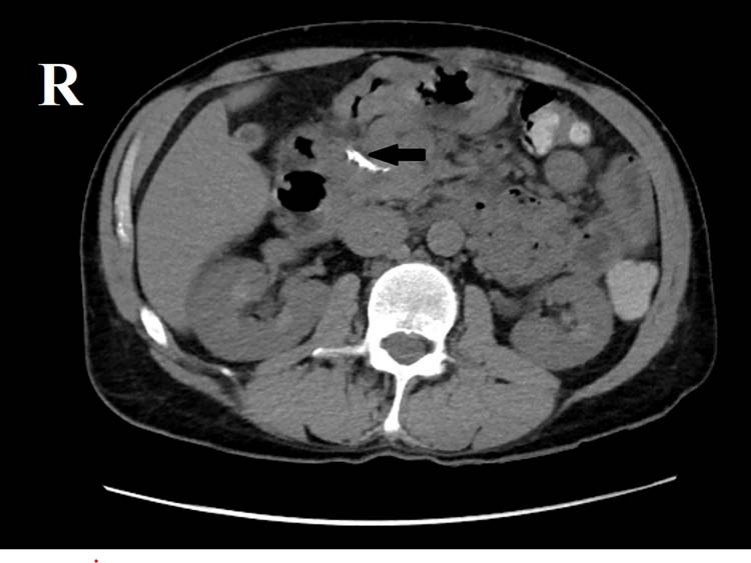
CT scan of pancreas (without contrast).

**Figure 3:**
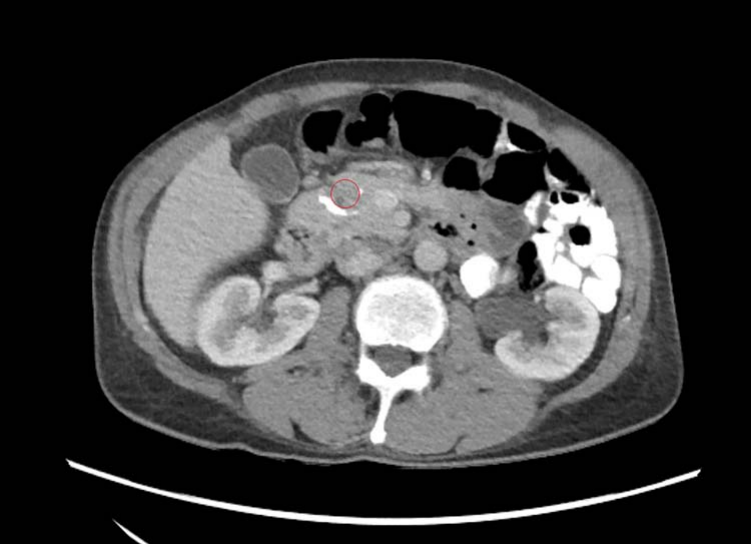
Contrast enhanced CT scan of pancreas.

**Figure 4:**
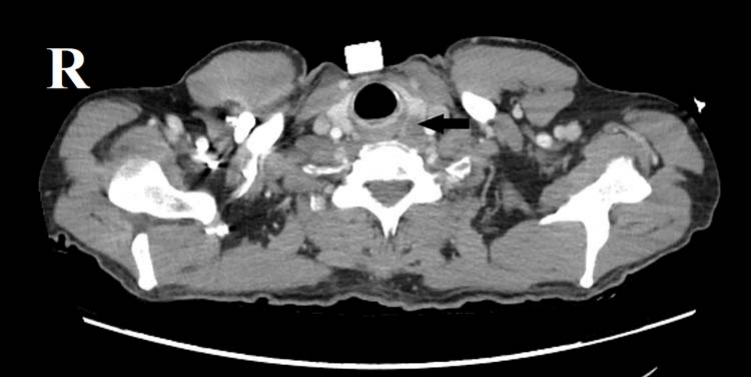
CT pulmonary angiogram.

Ultrasonography of the abdomen showed a calculus in the right renal pelvis measuring up to 2 cm. Multiple concretions and cysts were seen throughout both kidneys. Computed tomography (CT) scan of the abdomen (Fig. 1) showed right renal pelvis calculus measuring 1.5 × 1.3 cm. CT scan of pancreas (Fig. 2) revealed coarse calcification of 1.3 × 0.5 cm in the pancreatic head. Moreover, a contrast-enhanced CT (CECT) scan of the abdomen (Fig. 3) showed a slightly hypodense lesion of 1.1 × 0.4 cm circumscribed by calcification. Also, CT pulmonary angiogram was done to rule out pulmonary embolism, which incidentally showed (Fig. 4) a hypodense lesion 1.6 × 1.2 cm revealing an enlarged parathyroid gland. Hand X-ray was normal.

The patient was given intravenous normal saline for hypercalcaemia and vitamin D3 50 000 U for low vitamin D during the hospital stay. Also, intravenous proton pump inhibitors drip and sucralfate were given. Initially, the patient could only tolerate a liquid diet. He tolerated liquids to a solid diet during the treatment course. We advised for genetic testing of the patient and his first-degree relatives, and referred him to a tertiary care hospital for further evaluation and management.

## DISCUSSION

The diagnosis of MEN 1 syndrome can be made clinically based on family history and demonstration of MEN 1 association tumours or molecular diagnosis by genetic testing [2].

Primary hyperparathyroidism is the most common endocrinopathy associated with MEN 1 syndrome [3, 4]. It can have neuropsychiatric, gastrointestinal, musculoskeletal, renal and cardiovascular manifestations [1, 4]. The prevalence of MEN 1-associated gastrinomas is around 40%, with less than 10% occurring in the pancreas [3]. These patients can present with epigastric pain, chest pain and peptic ulcers [3, 4]. Our patient had nephrolithiasis and hypercalciuria, which points towards renal system involvement due to primary hyperparathyroidism. Moreover, dysphagia, abdominal pain, vomiting and diarrhoea point toward Zollinger Ellison syndrome (ZES). His initial laboratory investigations showed hypercalcaemia, elevated PTH level and hypergastrinemia. Also, a CT scan of the abdomen revealed nephrolithiasis and a hypodense lesion in the pancreas. The patient was on PPI before showing up at the hospital; so there is a chance that the gastrin level during our investigation is lower than the required value for the diagnosis of gastrinoma. These findings along with significant family history, point towards MEN 1 syndrome with suspicion of associated ZES. During multiple visits to the hospital, emphasis on family history was lacking, and the focus was more on symptomatic management. This led to a delay in the diagnosis of MEN 1 in this patient. If not adequately managed, there is an increased mortality in these patients. The approach for management includes medical therapy, surgical therapy and genetic counselling [1, 3, 4]. Vigilant monitoring with regular workups is required in these patients [5, 6].

Our case shows that a high index of clinical suspicion should be maintained in patients presenting with hyperparathyroidism and increased gastrin level, with particular emphasis on family history. In case of high clinical suspicion, appropriate endocrinological investigations should be done. These patients often require a multidisciplinary approach for adequate management and genetic counselling.
